# Clinical and non-clinical determinants of cervical cancer mortality: A retrospective cohort study in Lagos, Nigeria

**DOI:** 10.3389/fonc.2023.1105649

**Published:** 2023-02-16

**Authors:** Idris Olasunmbo Ola, Adeyemi Adebola Okunowo, Muhammad Yaqub Habeebu, Junmei Miao Jonasson

**Affiliations:** ^1^ Global Health Program, Institute of Medicine, Sahlgrenska Academy, Gothenburg University, Gothenburg, Sweden; ^2^ Departments of Clinical and Community Service, The Blue-Pink Center for Women’s Health, Lagos, Nigeria; ^3^ Department of Obstetrics & Gynaecology, College of Medicine University of Lagos, Lagos, Nigeria; ^4^ Department of Obstetrics & Gynaecology, Lagos University Teaching Hospital, Lagos, Nigeria; ^5^ Department of Radiotherapy, College of Medicine, University of Lagos, Lagos, Nigeria; ^6^ Department of Radiotherapy, Lagos University Teaching Hospital, Lagos, Nigeria; ^7^ Lead Oncologist, NSIA-LUTH Cancer Centre (NLCC), Lagos, Nigeria; ^8^ School of Public Health and Community Medicine, Institute of Medicine, Sahlgrenska Academy, Gothenburg University, Gothenburg, Sweden; ^9^ Department of Research and Development, Region Halland, Halmstad, Sweden

**Keywords:** cervical cancer, cancer mortality, determinants of mortality, Nigeria, cancer epidemiology, cancer control, women’s health, oncology

## Abstract

**Introduction:**

Cervical cancer (CCa) is the fourth most frequent and a common cause of cancer mortality in women, the majority of whom live in low- and middle-income countries. Data on CCa mortality and its determinants have been poorly studied in Nigeria, resulting in a paucity of information that can assist patient management and cancer control policy.

**Aim:**

The purpose of this study was to assess the mortality rate among CCa patients in Nigeria as well as the major factors influencing CCa mortality.

**Study design:**

Data from the medical records of 343 CCa patients seen at the Lagos University Teaching Hospital and NSIA-LUTH Cancer Center from 2015 to 2021 were used in a retrospective cohort analysis. The hazard ratios (HR) and confidence intervals (CI) associated with the exposure variables and CCa mortality were calculated using Cox proportional hazard regression.

**Results:**

The CCa mortality rate was 30.5 per 100 women-years after 2.2 years of median follow-up. Clinical factors such as HIV/AIDS (adjusted HR [aHR]: 11.9; 95% CI: 4.6, 30.4), advanced clinical stage (aHR: 2.7; 95% CI: 1.5, 4.7), and anemia at presentation (aHR: 1.8; 95% CI: 1.1, 3.0) were associated with a higher mortality risk, as were non-clinical factors such as age at diagnosis >50 years (aHR: 1.4; 95% CI: 1.0, 1.9) and family history of CCa (aHR: 3.5; 95%CI: 1.1, 11.1)

**Conclusion:**

CCa has a high mortality rate in Nigeria. Incorporating these clinical and non-clinical factors into CCa management and control policies may improve women’s outcomes.

## 1 Introduction

Cancer of the uterine cervix or simply cervical cancer (CCa) is one of the leading causes of cancer-related mortality in women and a major disease of global public health concern. A recent estimate shows that about three billion women over the age of 15 years are at risk of the disease which is currently the fourth most frequent malignancy affecting women worldwide, after breast, colorectal, and lung cancers (1, 2). Globally, CCa represents about 7% of all cancer types, with significant variation along socioeconomic divides ranging from 2.8% in countries with very high human development index (HDI) to 17.7% in those with low HDI (2).

Mortality from CCa closely follows its incidence pattern, being the fourth major cause of cancer-related deaths, accounting for 7.5% of all cancer mortality in 2018, and with a global average age at death of 59 years (2). The age-standardized mortality rate ranges from 3 per 100,000 women in high-income countries to 20 per 100,000 women in low and middle-income countries (LMICs) (2, 3).

Recently, in the United States and many European countries, the incidence has remained stable, and mortality declined by almost 1% annually, owing to increased attention to prevention and early detection services and increased access to evidence-based treatments (3, 4). Although China and India account for almost one-third of global CCa incidence and mortality (2), a progressive decline in mortality has also been reported, especially in India, mainly due to improvements in their CCa control and healthcare services (5). In contrast, CCa incidence and mortality rates in Sub-Saharan Africa have been steadily increasing over the last 10-25 years, with the annual increase ranging from 1.3% to 9.5% in some countries (6, 7), culminating in about 10.8% of cancer deaths between 2015 and 2019 (8).

Studies in sub-Sahara Africa have reported cumulative mortality during a 2–5-year period ranging from 65% to 68% (9–11). In Nigeria, the 5-year CCa mortality prevalence was estimated at 22.11 per 100,000 women (12) and was responsible for 14.8% of all cancer deaths among Nigerian women in 2018 (13).

Empirical evidence has shown several factors that influence mortality risk from CCa. Late-stage hospital presentation of CCa is very common in sub-Sahara Africa (9, 14–17), and has been reported to generally reduce the 5-year survival rate from 92% in the early-stage presentation to about 17% in the late-stage when metastases has occurred (18, 19). Sociodemographic factors like lack of formal education, rural residence, fear, misconceptions, misinterpretation, ignorance, and longer investigation time are some of the health-seeking and health systems variables predisposing to late stage CCa presentation and its attendant increased mortality chance (17, 20). Dietary and lifestyle practices such as alcohol use, cigarette smoking, high body mass index (BMI), use of other substances of abuse (21), as well as socioeconomic/demographic factors like age at diagnosis, family history of CCa in first degree relatives, history of abortion, and age at first marriage and birth have been described as determinants of CCa mortality (20, 22).

Other clinical factors such as anemia, HIV/AIDS, comorbidity, type of treatment received, and histological type of the disease have also been reported to influence CCa outcome (9–11, 22).

Although these factors have varying influences on CCa mortality risk, to our knowledge, their exact roles in determining CCa outcome among women in Nigeria have been poorly studied. Nigeria has a large population of CCa-at-risk women whose mortality has not been adequately investigated (13). Consequently, there is a scarcity of relevant data on CCa mortality rate and the factors that influence it (2, 23).

This study aimed to estimate the mortality rate and the clinical and non-clinical factors that determine mortality among CCa patients in Lagos, Nigeria.

## 2 Materials and methods

### 2.1 Study design and setting

A retrospective cohort study was conducted in the gynecological oncology unit of Lagos University Teaching Hospital (LUTH) and the NSIA-LUTH Cancer Center (NLCC) both in Lagos, Nigeria.

LUTH is an 800 bedded tertiary teaching and referral hospital in Lagos, Nigeria. Through a public-private partnership model, LUTH, in partnership with the NSIA Healthcare Development and Investment Company opened the NLCC, an advanced cancer treatment center that has synchronized cancer treatment services in LUTH since May 29, 2019 (24).

Medical records of CCa patients diagnosed between January 1^st^, 2015, and December 31st, 2021, were retrieved from the medical records department through manual sorting of all the gynecological cancer cases seen in LUTH and NLCC during the period. Retrieval of records was supported by trained staff of the medical record units of both the LUTH and the NLCC.

Records of participants who were newly diagnosed with CCa or referred from other hospitals on account of CCa, whose clinical diagnoses were ascertained with a histological report and attended the gyne-oncology unit of LUTH or the NLCC between January 1^st^, 2015, and December 31^st^, 2021, were included in the study.

#### 2.1.1 Exposure variables

Data were extracted from medical records using a pretested structured questionnaire. Socio-demographic, family, and social history data were used as non-clinical exposure variables, whereas clinical and histopathology data were used as clinical exposure variables.

Data on histology was derived from the histology report of pathologists. Three categories were derived based on the frequency of occurrence of the different histo-types: squamous cell carcinoma, adenocarcinoma, and other types including adenosquamous and small cell carcinoma.

Data on comorbidity was based on the Deyo modification of the Charlson Comorbidity Index after excluding cervical cancer (25).

Data on anemia was obtained using the patient’s packed cell volume (PCV) and classified according to WHO recommendations for non-pregnant women 15 years or older. Severe anemia was PCV <24%, moderate anemia 24–32.9%, mild anemia 33–35.9%, and PCV 36% or higher constitute no anemia (26).

Clinical staging of the disease was based on the International Federation of Gynecology and Obstetrics (FIGO) 2018 classification into early and late disease (27). For this study, we also explored further categorization into early (FIGO I—IIA), late (FIGO IIB—IIIC), and advanced disease (FIGO IVA and IVB) to observe more clearly the effect of these stages on CCa death. Treatment data were also obtained from the oncologists/surgeon’s management plan and the nursing report of medications and treatments administered for those that were admitted during their treatment. Data were classified based on whether the patient had chemotherapy, radiotherapy, or surgery only, and varying combinations of the three modalities.

Information on HIV/AIDS status was extracted from the laboratory investigation reports usually carried out as part of baseline investigations at hospital presentation.

Information about age, location/residence, occupation, religion, ethnicity, educational level, and marital status were extracted from the biodata section of the records. Age at diagnosis was categorized broadly as age 50 years or younger and age older than 50 years based on previous research (10). Data on occupation was classified as either unemployed or based on skill levels derived using the latest versions of the International Labor Organization’s International Standard Classification of Occupations 2008 [ISCO-08 and ISCO-88] (28). Participants who were engaged in some forms of occupation were sub-classified as high-skilled, medium-skilled, or low-skilled occupation, armed forces, or not elsewhere classified.

The family and social history section of the medical records provided information on the family history of CCa in a first-degree relative, cigarette smoking, alcohol intake, use of other substances, including herbal preparations, and parity, defined by the number of pregnancies carried to viability, irrespective of whether the child was alive or not.

#### 2.1.2 Outcome variable

Mortality during the follow-up period was the dependent, binary outcome variable, and was confirmed through the death register, death summary form, or phone calls to the next of kin of patients whose outcomes could not be ascertained through existing records. Empirical evidence has proven verbal confirmation of mortality to be accurate and reliable in resource-constrained settings with low coverage of vital registrations, including death registration (29).

Follow-up started from the first histological diagnosis of CCa and ended on the date of death of the participant, referral for the continuation of care outside LUTH or NLCC (often due to proximity to their location), or the common closing date on December 31^st^, 2021, whichever came first.

### 2.2 Statistical analysis

Descriptive analysis was conducted using frequencies, means, and percentages. Inferential statistics were obtained using the Cox proportional hazards regression model. Hazard ratios (HRs) and their confidence intervals were used as measures of association between CCa diagnosis and mortality.

Univariate analyses were conducted for all exposure/independent variables of CCa mortality using the log-rank tests of equality. Variables such as family history of cervical cancer, cigarette smoking, educational level, alcohol, and other substances use, HIV/AIDS status, anemia, stage of the disease, and types of treatment received with a p-value less than 0.25 (30) were considered relevant and were included in the multivariable Cox regression model. In the multivariable analysis, the p-value of anemia was statistically insignificant (p-value = 0.856), however, we retained anemia in the model because the overall p-value for the model was <0.001 and previous research showed that anemia was relevant in the model (9, 10).

Proportionality assumption was checked using the Schoenfeld and scaled Schoenfeld residuals and the goodness of fit of the final model was checked using the Cox-Snell residuals plotted in a Nelson-Aalen cumulative hazard function.

All statistical tests were 2-tailed, and type-1 error was set at 0.05 level of significance. The power of the study was 80%. Missing data were handled using complete case analysis for each variable of interest and the low level of missing data increased the validity of the results.

### 2.3 Ethical considerations

Ethical approval for study was granted by the ethical review committee of LUTH with approval number ADM/DSCST/HREC/APP/4939. This approval was used to gain permission from the gyne-oncology unit and the medical records department to access the medical records. A secondary approval was also granted by the administrative boards of NSIA-LUTH Cancer Center to access medical records in the center.

## 3 Results

Out of 420 cases of CCa seen during the study period, a total of 343 (81.7%) women with complete records were included in the analysis.

### 3.1 Non-clinical characteristics of women with cervical cancer

The mean age at CCa diagnosis (entry) was 55.3 years (SD 12.5 years) with age range between 28 and 88 years. The age group 55–64 years recorded the highest number of cases of CCa during the study period.

Two-thirds of the study population were suburban residents, and more than one-thirds had a secondary level of education. Fifty-six (16.6%) were unemployed and about 55% (185) were engaged in various low-skill occupations. Approximately 25% (65) had a history of substance use, with alcohol intake responsible for the highest substance use at 17.6% (57) while only about 5% (16) smoked cigarette (**Table 1**). Other non-clinical characteristics are described in **Table 1**.

**Table 1:** Non-clinical characteristics of the study population including results of univariate analysis.

Variables	Categories	Frequency N (%)	Total CCa Mortality N (%)	p-value in univariate analysis (log-rank test)(<0.25)
Age (years)	50 years or less >50 years Missing	119 (34.7) 214 (62.4) 10 (2.9)	70 (58.8) 132 (61.7) 0	0.0337* **^#^**
Ethnicity	Yoruba Igbo Others Missing	172 (50.2) 85 (24.8) 86 (25) 0	108 (62.8) 47 (55.3) 47 (54.7) 0	0.3207
Residence	Rural Suburban Urban Missing	9 (3.5) 158 (61.2) 91 (35.3) 85 (24.8)	3 (33.3) 102 (64.6) 56 (61.5) 0	0.3953
Education	Primary Secondary Tertiary No formal education Missing	78 (24.8) 106 (33.6) 75 (23.8) 56 (17.8) 28 (8.2)	47 (60.3) 61 (57.5) 37 (49.3) 37 (66.1) 0	0.0173*
Occupation	Unemployed High-Skill Occupation Medium-Skill Low-Skill Occupation Armed forces Unclassified Missing	56 (16.6) 32 (9.5) 54 (16.0) 185 (54.9) 2 (0.6) 8 (2.4) 6 (1.7)	34 (60.7) 15 (46.9) 33 (61.1) 109 (58.9) 1 (50) 5 (62.5) 0	0.4003
Religion	Islam Christianity Missing	74 (21.6) 269 (78.4) 0	46 (62.2) 156 (58.0) 0	0.6590
Marital Status	Single Married Missing	7 (2.2) 307 (97.8) 29 (8.5)	5 (71.4) 181 (59.0) 0	0.5130
Parity	0-3 4-7 8-11 >11 Missing	71 (23.5) 169 (56.0) 61 (20.2) 1 (0.3) 41 (12)	42 (59.2) 100 (59.2) 36 (59.0) 0 (0.0) 0	0.9256
Family History of CCa	Yes No Missing	5 (1.6) 313 (98.4) 25 (7.3)	4 (80.0) 190 (60.7) 0	0.0210* **^#^**
Cigarette smoking	Cigarette Smoking No smoking Missing	16 (5.0) 301 (87.8) 26 (7.6)	13 (81.3) 185 (61.5) 0	0.0469*
Alcohol	Alcohol intake No alcohol intake Missing	56 (17.7) 261 (82.3) 26 (7.6)	40 (71.4) 158 (60.5) 0	0.0794*
Other substances use	Yes No Missing	11 (4.4) 240 (95.6) 83 (24.2)	11 (100) 148 (61.7) 0	0.0207*
Overall treatment outcomes	Alive Dead Referred Loss to follow-up Missing	33 (9.9) 202 (59) 35 (10.5) 62 (18.6) 10 (2.9)		

*Statistically significant variables in univariate analysis that were included in multivariate analysis.

# Variables significantly associated with CCa mortality in multivariate analysis (p < 0.05).

### 3.2 Clinical characteristics of women with cervical cancer

A total of 202 (59%) CCa deaths were recorded during the follow-up period with a mean age of 57.6 years (SD 13.0 years) at death. Nearly 10% (33) of the study population was still alive, and the outcome could not be ascertained in over one-quarter of the study population due to either referral to other centers, short-term management at LUTH and NLCC, 35 (10%) or loss to follow-up 62 (18.6%). Attempts to contact these women and their relatives *via* phone call were not successful.

Of the cases reviewed, 245 (86.3%) presented in late stages (FIGO stage IIB-IVB) and about 5% (15) were diagnosed with HIV/AIDS during treatment. Squamous cell carcinoma accounted for over four-fifths (82.6%) of all CCa histologic types seen in the cohort. Over 83% (241) of the study participants were anemic, approximately a quarter of these women had severe anemia. Less than half [45.4%, (117)] of the participants had comorbid conditions and most patients in the cohort 149 (55.6%) received combination therapy involving chemotherapy and radiotherapy. (**Table 2**).

**Table 2:** Clinical characteristics of the study population, mortality pattern within each group, and results of univariate analysis.

Variables	Categories	Frequency N (%)	CCa Mortality N (%)	p-value in univariate analysis (log-rank test) (<0.25)
Clinical Stage	Early Advanced/Late Late (IIb-IIIc) Very Advanced (>=IVa) Missing	39 (13.7) 245 (86.3) 167 (58.8) 78 (27.5) 59 (17.2)	15 (38.5) 159 (64.9) 96 (59.6) 63 (82.9) 0	0.0002* **^#^**
Anemia	No anemia Anemia Mild Moderate Severe Missing	48 (16.6) 241 (83.4) 50 (17.4) 131 (45.5) 59 (20.5) 54 (15.7)	18 (37.5) 164 (68.0) 28 (56.0) 93 (71.0) 42 (71.2) 0	0.0247* **^#^**
Histology	Squamous Adenocarcinoma Adenosquamous Others Missing	263 (83) 34 (10.7) 8 (2.5) 12 (3.8) 26 (7.6)	152 (57.8) 22 (64.7) 4 (50) 6 (50) 0	0.5224
HIV/AIDS	Negative Positive Missing	300 (95.2) 15 (4.8) 28 (8.2)	181(60.3) 12(80.0) 0	0.0000* **^#^**
Comorbidity	No Yes Missing	141(54.7) 117 (45.3) 85 (24.8)	91(64.5) 67(57.3) 0	0.5282
Treatment	Chemotherapy Radiotherapy Surgery Chemo+Radio Surgery+Radio Surgery+Chemo Surgery+Radio+Chemo Missing	30 (11.2) 45 (16.8) 9 (3.4) 149 (55.6) 4 (1.5) 5 (1.9) 26 (9.7) 75 (21.9)	22 (73.3) 28 (62.2) 5 (55.6) 90 (60.4) 0 (0.0) 3 (60.0) 10 (38.5) 0	0.1160*

*Statistically significant variables in univariate analysis that were included in multivariate analysis.

# Variables significantly associated with CCa mortality in multivariate analysis (p < 0.05).

### 3.3 Cervical cancer mortality

The crude all-cause mortality rate was 30.5 per 100 person-years with 2.2 years median follow-up time, corresponding to a cumulative mortality of 59%.

Relative disparities in CCa mortality were most pronounced for very advanced stage disease (FIGO IVA and IVB) with only 17.1% survival after 7 years of diagnosis, which increased by almost four-folds (61.5%) when diagnosis was made at early stages (FIGO I and IIA). Mortality was lowest [10 (40%)] among those that received three combination therapies involving surgery, chemotherapy, and radiotherapy, but highest among those that received either chemotherapy alone [22 (73.3%)] or in combination with surgery [3 (75%)]. Histologically, adenocarcinoma had the lowest proportional survival from the disease 11(33.3%). Approximately nine in 10 CCa mortality had some form of anemia and HIV/AIDS was responsible for a disproportionately higher mortality (85.7%) compared to 62% among those without HIV/AIDS. (**Table 2**).

### 3.4 Non-clinical determinants of cervical cancer mortality

In the analysis of non-clinical determinants of CCa mortality, the gradient was especially noticeable in patients who had family history of CCa in a first degree relative, with more than four-folds increase in risk of dying from the disease (Age-adjusted hazard ratio [aHR]: 4.1, 95% CI: 1.8, 10.0). Being older than 50 years of age at diagnosis was also associated with a 40% higher chance of dying from CCa than those who presented at 50 or younger age (HR: 1.4, 95% CI: 1.0, 1.9). (**Table 3**)

**Table 3:** Age-adjusted and unadjusted hazard ratios for CCa mortality.

Variables	Categories	Unadjusted HR (95% CI)	p-value	Age-adjusted HR (95% CI)	p-value
Clinical Determinants					
HIV/AIDS	Negative Positive	1.0 2.6 (1.4, 4.7)	0.002	1.0 2.7 (1.5, 4.8)	0.001
Clinical Stage	Early Late/Advanced Early (I-IIA) Late stage (IIB-IIIC) Advanced (IVA-IVB)	1.0 2.8 (1.6, 4.8) 1.0 2.4 (1.4, 4.2) 3.7 (2.1, 6.8)	<0.001 0.003 <0.001	1.0 2.7 (1.5, 4.7) 1.0 2.3 (1.3, 4.0) 3.7 (2.1, 6.7)	<0.001 0.005 <0.001
Anemia	No anemia Anemia Mild Moderate Severe	1.0 1.7 (1.1, 2.8) 1.0 1.8 (1.2, 2.8) 2.4 (1.5, 3.9)	0.027 0.006 <0.001	1.0 1.8 (1.1, 3.0) 1.0 1.8 (1.2, 2.8) 2.4 (1.4, 3.9)	0.017 0.006 0.001
Non-Clinical Determinants					
Age	≤50 years >50 years	1.0 1.4 (1.0, 1.9)	0.035	1.0 1.4 (1.0, 1.9)	0.035
Family History of CCa	No Yes	1.0 3.5 (1.1, 11.1)	0.031	1.0 4.1 (1.8, 13.0)	0.017

### 3.5 Clinical determinants of cervical cancer mortality

When clinical stage of the disease at diagnosis was explored, clinical presentation at late or advanced stages (FIGO IIB-IVB) correlated with a three-folds increase in mortality risk (aHR: 2.7, 95% CI: 1.5-4.7). On further analysis, there was a sharp upward gradient in the risk from the baseline early stage (FIGO I-IIA) disease to advanced (FIGO IVA-IVB) disease. (**Table 3**)

The chances of CCa death rose by almost three-folds when a CCa patient had underlying or recently diagnosed HIV/AIDS disease (aHR: 2.7, 95% CI: 1.5, 4.8). Anemia conferred approximately 80% increased risk of CCa mortality (aHR: 1.8, 95% CI: 1.1, 3.0). However, the pattern observed along the levels of anemia was similar to that of clinical stage of the disease, with a steep upward gradient from mild to severe. (**Table 3**)

## 4 Discussion

This study investigated the clinical and non-clinical factors that influence mortality from CCa in Nigeria. The cumulative prevalence of CCa mortality over the 7-year period was 59%, with an all-cause mortality rate of 30.5 per 100 women-years and a median follow-up time of 2.2 years. The risk of CCa mortality increases with clinical factors such as anemia, advanced clinical stage of CCa at presentation, and coexisting HIV/AIDS. Few non-clinical factors including family history of CCa in a first-degree relative and older age over 50 years at diagnosis were associated with increased mortality risk.

The mortality rate in this study is substantial, but very strongly correlated with the recent estimate of 31 per 100-women years in Ethiopia (9). However, it also varies widely from estimates in other previous studies with the rates ranging from 15.6 to as high as 79.8 per 100 women-years (10, 11). This may be due to the selection of the study population as mortality from hospital-based data are usually higher than that of the population. Similarly, variations in the sample size, study period, and focus of the study may also contribute to the wide variation observed (11). Another factor that could possibly explain the disparities is the poor and inconsistent record of mortality in most LMICs especially sub-Saharan Africa where national vital statistics including death registers are grossly inadequate or absent (31). Thus, the reported mortality rates were entirely dependent on how much of the mortality and outcome data could be retrieved by various researchers.

Overall, most of the factors revealed in this study corroborate the findings from previous studies (9–11, 23), but also find many of the previously documented factors insignificant in our study cohort.

Anemia was found in over four-fifths of CCa patients in our study. This estimate was significantly higher relative to earlier reports in Ethiopia (51.2%), Kenya (56.5%), Northern Nigeria (51%) and Korea (36.4%) (11, 14, 32, 33). In the United States, however, Hufnagel and colleagues (34) found up to 64% of gynecologic cancer (including CCa) patients developed anemia within six months of their diagnosis while the European Cancer Anemia Survey (ECAS) found up to 68% prevalence of anemia among European cancer patients during a 6-month survey period (35).

Anemia in cancer patients has a complex etiopathogenesis. The probable contributing causes include bleeding, dietary inadequacies, hemolysis, diminished erythropoietin levels, inflammation with increased hepcidin activity, and chemotherapy toxicity in marrow precursors (for those already commenced on chemotherapy) (36). In addition to population characteristics, stage of disease, and treatment modality received, presence of other comorbidities could significantly influence the incidence and severity of anemia, leading to considerable variation in its estimation (37).

In Nigeria, the rate of anemia among healthy women of reproductive age is high and it varies widely across regions ranging between 21% and 55% according to recent estimates (38, 39). This high pre-morbid anemia among women increases the risk of symptomatic anemia when cancer sets in, thus further explaining the high prevalence observed in our study. Of note is that most of the previous studies used a lower PCV cutoff value of 30% as normal blood levels (11), contrary to the WHO-recommended 36% for non-pregnant, reproductive-aged women (26). Most clinicians and researchers in Nigeria use the 30% cutoff for the general population because most people are asymptomatic at that level (40, 41).

Anemia at presentation in cancer patients could be due to anemia of chronic disease (ACD) which are often exacerbated by chronic blood loss and nutritional deficiencies, especially Iron deficiency (42). ACD is caused by a malfunction in Iron metabolism induced by excessive release of the Iron transport modulator hepcidin, resulting in a paradoxical Iron deficiency and anemia (43).

Our study shows that anemia significantly reduces disease-specific survival by more than 80%. A possible mechanism responsible for this is anemia-induced hypoxia which is associated with decreased tumor susceptibility to anticancer medications and increased morbidity in general (10, 44).

Over 86% of our study cohort were diagnosed at advanced stage of the disease. This is close to the East African estimates in Kenya and Ethiopia (14, 15). However, this estimate is significantly higher than the global average of about 60% and the African average of 62% (20). It also differs from other estimates such as in Nepal (65.5%), Northern Nigeria (72.3%), Ethiopia (60.4), and Brazil (70.6%) (11, 16, 45, 46).

The stage of CCa at diagnosis could partly reflect the accessibility and uptake of cancer screening services (47). Generally, most LMICs lack efficient national cancer screening and control programs that cover a substantial proportion of eligible women. In Nigeria, only 0.7–8% of reproductive-age women had been screened at least once in their lifetime (48, 49) similar to findings in Ghana (2.4%) but contrary to findings in Uganda (20.6%), and Malawi (40.2%) (50–52).

Healthcare system inadequacies could also be a significant contributor to late-stage diagnosis of CCa. For example, the prevalence of misdiagnosis especially among the cases referred from private health facilities was reported to be as high as 87.6%, and prolonged investigation time and degree of secondary (facility-based) delays could be responsible for delay in early diagnosis and commencement of appropriate care (17).

Our study showed that late-stage presentation was significantly associated with a higher chance of mortality, and this agrees with previous reports (18, 19). The mechanism of this effect is multifactorial, and probably due to multi organs involvement, distant spread to multiple vital organs, obstructive effect on the urinary system leading to kidney disorders, increasing risk of severe anemia, and overall higher morbidity.

HIV/AIDS confers significantly increased mortality risk up to three-folds among CCa patients. Similarly, other findings have reported a 2 to 3-fold increase in the risk of mortality among CCa patients living with HIV/AIDS (9, 53).

The association of HIV/AIDS with immunosuppression has been shown to cause poor clearance of high-risk HPV oncotypes and facilitate its infection of the cervical basal cells (54). These mechanisms enhance susceptibility of HIV patients to CCa. However, its effect on survival from CCa is complex and still insufficiently understood. Current understanding shows that HIV primarily impairs the ability of the cellular immune system to clear cancerous cells after treatment and in fact, could directly lead to poorer response to CCa therapy (53, 55). In other words, it leads to increased susceptibility to early cancer recurrence and severity. A recent study in Brazil showed that HIV infection did not affect initial treatment response or early mortality in women with cervical cancer, but it did increase relapse after achieving a complete response or remission, and late mortality (56). Similarly, the immunosuppression linked with HIV/AIDS also increases the susceptibility of CCa patients to other life-threatening AIDS-defining infections and cancers (57) which adds significant morbidity and increase their mortality risk.

In our study, about 5% of the patients had HIV/AIDS compared to previous studies that reported approximately 8% incidence (9, 11). This is a substantially higher HIV/AIDS prevalence than the recent national estimate of 1.4% in Nigeria (58), showing the higher propensity of women living with HIV/AIDS to develop CCa.

The mean age at diagnosis and death in our study is consistent with reported global estimates (2) and a large population-based estimate from an African study (59). These mean ages are, however, slightly higher at diagnosis and lower at mortality than the mean ages reported in the United States and Europe (3, 4).

As shown in our study, and in consonance with previous studies, being older than 50 years at time of diagnosis, was associated with a substantially higher risk of death from CCa (9, 10). However, no consistent pattern was observed when 13 African countries were studied (57). Conversely, age at diagnosis was found to be protective in an Ethiopian study (15), however, the cohort used for the study had younger women with mean cohort age of 49 years which might have impacted the study finding.

A risk level of about 46% has been reported among the age group 50–69 years, including an almost three-fold higher risk among CCa patients older than 70 years (60). This is consistent with the findings in our study.

Age has multiple effects on CCa outcome, and it is an independent negative prognostic factor irrespective of clinical stage, ethnicity or histological type of the disease (60). Older women are more likely to receive less-aggressive treatments (60) such as surgery and chemotherapy because of concern for their overall tolerability of stress of surgery or the ability of their organs to effectively metabolize anticancer medications. Older age also correlates with an increasing risk of other comorbidities and anemia which increases the morbidity and mortality from the disease (61).

The most common understanding of the role of family history in CCa is the increased risk of occurrence of the disease usually at genetic level. However, our study suggests that family history of CCa might also play a role in the outcome of CCa. A time-to-death analysis conducted among Ethiopian women supported this finding (22). However, caution should be applied in interpreting this finding due to the relatively small number of patients with family history of CCa in our data. It is also important to note that all the patients in our study who had family history of CCa presented at advanced stage mostly stage IVA or IVB. This could have significantly influenced the findings observed.

The biological mechanism of effect of family history on CCa mortality is still unclear as no previous descriptions were found in the literature at the time of reporting this study.

### 4.1 Study strengths and limitations

This study’s categorization of the factors into clinical and non-clinical determinants helps to put further clarity on the type of interventions that would be appropriate to address the factors. The study was based on relatively accurate and reliable data collected by trained health personnel and with high consistency because of multiple reviews by more than one medical personnel during management of the patients. We also achieved a relatively high complete follow-up rate of about 71% of the study population and were able to compile comprehensive information on the variables despite the prevailing challenges to record keeping, retrieval, data collection and follow-up in our environment. The strict reliance on histological diagnosis of CCa conferred high level of diagnostic accuracy and consistent determination of the follow-up time.

In the evaluation of women with family history of CCa in first-degree relatives, the power of the study was limited due to the small number of women who had family history of the disease. Being an institution-based study data, its interpretation may also partly reflect on institutional factors and may be difficult to completely extrapolate the findings to the general population. The lack of a national or regional database of death records is a major impediment for studies on cancer mortality in many LMICs, as observed in our study. The results of our study could have been bolstered even further by the confirmation of every death through such a database.

## 5 Conclusions

The study of clinical and non-clinical factors influencing cervical cancer mortality in Lagos, Nigeria, shows that mortality rate from the disease is very high. Clinical factors such as presence of anemia, HIV/AIDS, and advanced clinical stage disease, in addition to non-clinical factors like age above 50 years at diagnosis and family history of the disease in a first-degree relative were associated with higher CCa mortality risk. Consideration of these clinical and non-clinical factors during CCa management and control may improve the outcome for Nigerian women.

## Data availability statement

The raw data supporting the conclusions of this article will be made available by the authors, without undue reservation.

## Ethics statement

Ethical approval for study was granted by the ethical review committee of Lagos University Teaching Hospital (LUTH) with approval number ADM/DSCST/HREC/APP/4939. Written informed consent for participation was not required for this study in accordance with the institutional requirements

## Author contributions

IO conceptualized the topic and research methodology, conducted data analysis, and prepared the first drafts of the manuscript and subsequent co-authors inputs. AO was involved in data curation and provided supervision. MH was involved in data curation. IO, AO, and MH were involved in securing ethical approval for the study. JM provided supervision. All authors contributed to the article and approved the submitted version.
